# Intricate Regulatory Mechanisms of the Anaphase-Promoting Complex/Cyclosome and Its Role in Chromatin Regulation

**DOI:** 10.3389/fcell.2021.687515

**Published:** 2021-05-24

**Authors:** Tatyana Bodrug, Kaeli A. Welsh, Megan Hinkle, Michael J. Emanuele, Nicholas G. Brown

**Affiliations:** ^1^Department of Biochemistry and Biophysics, Lineberger Comprehensive Cancer Center, University of North Carolina at Chapel Hill, Chapel Hill, NC, United States; ^2^Department of Pharmacology, Lineberger Comprehensive Cancer Center, University of North Carolina School of Medicine, Chapel Hill, NC, United States

**Keywords:** ubiquitin, cell cycle, chromatin, structural biology, ubiquitin ligase (E3), cryo-EM, Anaphase-Promoting Complex/Cyclosome

## Abstract

The ubiquitin (Ub)-proteasome system is vital to nearly every biological process in eukaryotes. Specifically, the conjugation of Ub to target proteins by Ub ligases, such as the Anaphase-Promoting Complex/Cyclosome (APC/C), is paramount for cell cycle transitions as it leads to the irreversible destruction of cell cycle regulators by the proteasome. Through this activity, the RING Ub ligase APC/C governs mitosis, G1, and numerous aspects of neurobiology. Pioneering cryo-EM, biochemical reconstitution, and cell-based studies have illuminated many aspects of the conformational dynamics of this large, multi-subunit complex and the sophisticated regulation of APC/C function. More recent studies have revealed new mechanisms that selectively dictate APC/C activity and explore additional pathways that are controlled by APC/C-mediated ubiquitination, including an intimate relationship with chromatin regulation. These tasks go beyond the traditional cell cycle role historically ascribed to the APC/C. Here, we review these novel findings, examine the mechanistic implications of APC/C regulation, and discuss the role of the APC/C in previously unappreciated signaling pathways.

## Introduction

The post-translational modification of cellular proteins with ubiquitin (Ub) is a predominant form of eukaryotic regulation (Rape, 2018). Since the initial discoveries of Ub-dependent processes, there was an intimate link between the role of Ub in the cell cycle and the regulation of chromatin (Goldknopf et al., 1975; Matsui et al., 1979; West and Bonner, 1980; Irniger et al., 1995; King et al., 1995, 1996; Sudakin et al., 1995; Yamano et al., 1996; Robzyk et al., 2000). In the 1990s, the cell cycle and Ub fields were significantly advanced by the discovery of Ub-dependent protein turnover of cycling proteins (Yamano et al., 1996). Specifically, cullin-RING Ub ligases, SCFs (SKP1–CUL1–F-box protein) and the Anaphase-Promoting Complex/Cyclosome (APC/C) drive the cell cycle by tagging key regulators with polyubiquitin chains, resulting in their destruction by the proteasome (King et al., 1996; Peters, 1998). Changes in chromatin architecture have also been tightly linked to the cell cycle (Kouzarides, 2007; Bannister and Kouzarides, 2011; Struhl and Segal, 2013). Chromatin modifications, such as acetylation, methylation, and ubiquitination, are important contributing factors in mediating changes of key cell cycle regulators at the transcriptional level (Whitfield et al., 2002; Kouzarides, 2007; Bar-Joseph et al., 2008; Bannister and Kouzarides, 2011; Grant et al., 2013; Pena-Diaz et al., 2013; Breiling and Lyko, 2015). Recent developments have shown a link between transcriptional processes involving chromatin modification and protein turnover, including a role for E3 ligases such as the APC/C.

The 1.2 MDa APC/C is a molecular machine required for the cell cycle in all eukaryotes (Alfieri et al., 2017; Watson et al., 2019a). Polyubiquitination by the APC/C is responsible for the degradation of several substrates, e.g., Securin and Cyclin B, and is selectively regulated by a variety of factors (Irniger et al., 1995; King et al., 1995, 1996; Lahav-Baratz et al., 1995; Sudakin et al., 1995; Tugendreich et al., 1995; Aristarkhov et al., 1996; Yamano et al., 1996; Yu et al., 1996). This regulation is at the heart of the G1/S transition, mitotic checkpoint, and genome stability; consequently, APC/C dysregulation is common in cancer (Lukas et al., 1999; Garcia-Higuera et al., 2008; Kim and Yu, 2011; Cappell et al., 2016; Choudhury et al., 2017; Sansregret et al., 2017; Wan et al., 2017). The APC/C is a multisubunit Ub ligase with several moving parts, numerous substrates, and is involved in a number of non-mitotic processes (Konishi et al., 2004; Eguren et al., 2011; Kim and Yu, 2011; Primorac and Musacchio, 2013; Davey and Morgan, 2016; Huang and Bonni, 2016; Alfieri et al., 2017; Bakos et al., 2018; Watson et al., 2019a). However, new mechanisms of substrate recruitment and their subsequent ubiquitination have continued to be identified along with novel substrates. Here, we will review several recent studies of APC/C-dependent ubiquitination and examine how the APC/C is at the nexus of both the cell cycle and chromatin biology.

## The Ubiquitin System

**FIGURE 1:**
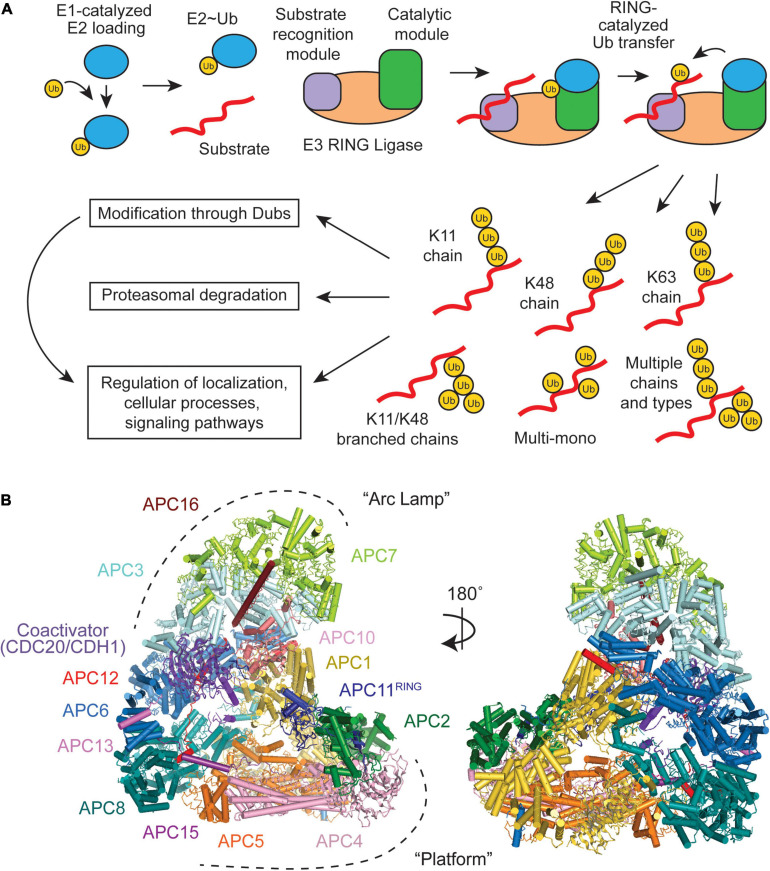
Overview of a RING E3 Ub ligase mechanism and structural overview of the Anaphase-Promoting Complex/Cyclosome (APC/C). **(A)** Substrate ubiquitination occurs through an E1-E2-E3 cascade, with RING E3s containing both a receptor for recognizing substrates and catalytic domains that facilitate Ub transfer. Multiple E2 binding events result in an array of Ub chain types that have specific downstream effects. **(B)** The APC/C consists of 19 polypeptides, which can be broken down into two large subdomains, the “Arc Lamp” and the “Platform” (PDB ID code 5G04) (Zhang et al., 2016).

An intricate set of enzymes serve as writers (E1-E2-E3 cascade), erasers (deubiquitinases), and readers (proteins that recognize Ub) of the Ub system (Figure 1A). E3 Ub ligases collaborate with E2s to decorate substrates with Ub, creating the Ub code (Komander and Rape, 2012; Yau and Rape, 2016; Haakonsen and Rape, 2019). E3s can be separated into three classes- RING (really interesting new gene), HECT (homologous to E6AP C-terminus), and RBR (RING-between-RING) (Metzger et al., 2014; Streich and Lima, 2014). Each E3 class uses a unique mechanism to transfer Ub from the E2 to the substrate. HECTs and RBRs accept the Ub from the E2, forming a covalent E3∼Ub conjugate (∼ denotes the covalent intermediate), and then transfer the Ub to the substrate (Buetow and Huang, 2016; Dove and Klevit, 2017). RINGs co-recruit the substrate and the E2, and facilitate the transfer of Ub from the E2 directly to the substrate (Buetow and Huang, 2016). Deubiquitinases (DUBs) fine-tune the Ub code by editing or removing the Ub chains (Mevissen and Komander, 2017). The edited Ub code is ultimately read by effector proteins that alter the polyubiquitinated target’s half-life, cellular localization, and enzymatic activity, depending on the Ub signal (Komander and Rape, 2012; Yau and Rape, 2016; Haakonsen and Rape, 2019). These diverse proteins can vary widely in both their enzymatic mechanisms and Ub-linkage specificities. It is the successful integration of E2s (∼40), E3 ligases (∼600), DUBs (∼100), and countless readers that result in a plethora of signaling outcomes that flow from the ubiquitin system.

During various cellular processes, a vast array of Ub chains can be formed because of the numerous amino groups on the protein target and on the Ub (M1, K6, K11, K27, K29, K33, K48, and K63) previously linked to the target (Figure 1A; reviewed in Komander and Rape, 2012; Yau and Rape, 2016; Clague et al., 2019; Griewahn et al., 2019; Haakonsen and Rape, 2019; Mattern et al., 2019). These polyubiquitin chains can either be homotypic, composed of a single chain type (e.g., K48), or heterotypic, containing mixed or branched linkages. Mixed chains are comprised of at least two different chain types (e.g., K11 and K48), but each Ub monomer is only modified at a single lysine site. In branched chains, a Ub monomer is modified at two or more lysine sites (e.g., K11/K48). These complex chains and topologies can be regarded as a code, because the linkage type dictates the fate of the substrate and cellular outcome. Different substrates are polyubiquitinated with different Ub linkage types. Some of the Ub tags are used for substrate degradation by the proteasome, whereas others are non-degradative. Often, the biological function and enzymes involved in forming heterotypic branched chains are unknown, even though 10–20% of polymerized Ub are modified at two or more lysines (Swatek et al., 2019).

## APC/C Fundamentals

The APC/C itself forms multiple types of Ub linkages, e.g., K11, K48, K63, and K11/K48 branched chains, and can monoubiquitinate its substrate target using a complex mechanism involving the E2s UBE2C and UBE2S (Aristarkhov et al., 1996; Yu et al., 1996; Kirkpatrick et al., 2006; Garnett et al., 2009; Williamson et al., 2009; Wu et al., 2010; Dimova et al., 2012; Meyer and Rape, 2014; Brown et al., 2016; Yau et al., 2017). Deciphering the structural organization and basic mechanisms of the APC/C and its E2s was made possible through two decades of work involving x-ray crystallography, native mass spectrometry, NMR, and cryo-Electron microscopy and relied on characterization of both human and yeast APC/C. Through advances in these techniques and our reconstitution capabilities of the complex assemblies that make up the APC/C, a detailed view of APC/C structure and function has emerged, with numerous aspects of the APC/C ubiquitination mechanisms uncovered through the combination of careful mutagenesis studies and artificially cross-linked intermediates.

The APC/C consists of 19 subunits, four of which are homodimers, that can be divided into two subcomplexes (Figure 1B). The “Platform” (APC1, 2, 4, 5, 8, 11, and 15) contains the catalytic core (APC2 and APC11) and the “Arc Lamp” (APC3, 6, 7, 10, 12, 13, and 16) provides a scaffolding element and a binding site for the substrate receptor/coactivator. Within the Arc Lamp, the subunits APC7, APC3, and APC6 are each dimers made up of TPR domain repeats (King et al., 1995; da Fonseca et al., 2011; Uzunova et al., 2012; Frye et al., 2013; Chang et al., 2014). Together, these subcomplexes work to facilitate and fine tune the APC/C’s highly dynamic ubiquitination activities.

**FIGURE 2:**
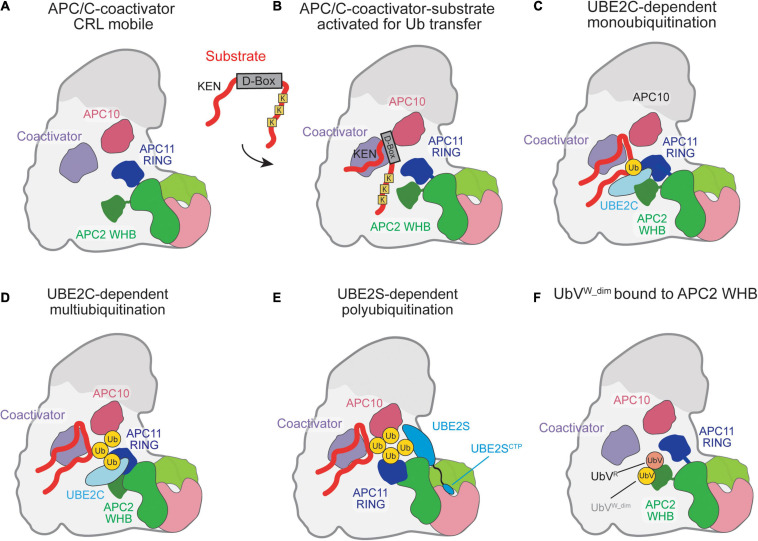
Schematic of ubiquitination reaction mechanisms catalyzed by the APC/C. **(A)** Coactivator-bound APC/C with the raised CRL being activated for Ub transfer as the APC2 WHB and APC11 RING domains are highly mobile. **(B)** Substrate recognition is mediated by the coactivator and APC10 which recognize the D-box on substrates. Substrate lysines are positioned near the E2 binding sites for Ub transfer by the RING. **(C)** UBE2C is clamped in place by the APC2 WHB and APC11 RING and positioned for catalysis of Ub transfer by the APC11-RING. **(D)** Multiple rounds of UBE2C∼Ub binding result in multiubiquitination. **(E)** UBE2S catalytic domain binds to APC2. The UBE2S CTP assists in UBE2S recruitment and activation of the APC/C catalytic domains by docking to the APC2/4 groove. **(F)** A cryptic Ub-binding site on the APC2 WHB amplifies recruitment of Ub to the APC/C, with a preference for K48-linked chains.

The recruitment of substrates to the APC/C and the positioning of its catalytic domains for ubiquitin transfer occur simultaneously through the binding one of two APC/C-specific coactivators, CDC20 or CDH1 (Visintin et al., 1997; Kraft et al., 2005; Buschhorn et al., 2011; da Fonseca et al., 2011; Chang et al., 2015). Binding of either coactivator to the APC/C occurs through sequences on their flexible N- or C-terminal domains, with C-terminal Ile-Arg motifs (IR tail) that bind on APC3, and an N-terminal C-box motif recognized by APC8 (Kimata et al., 2008; Matyskiela and Morgan, 2009; Chang et al., 2014; Yamaguchi et al., 2015). Also important for substrate recognition is APC10, which also contains an IR tail that binds to the second APC3 dimer and was found to be important for both substrate recognition and processivity (Buschhorn et al., 2011; da Fonseca et al., 2011; Chang et al., 2014). The coactivators thus provide binding sites for recruited substrates by recognizing ABBA and KEN box motifs on substrates (reviewed in Davey and Morgan, 2016) or working in conjunction with APC10 to bind D-box sequences (Figures 2A,B; Buschhorn et al., 2011; da Fonseca et al., 2011; Chang et al., 2014). The binding of coactivators is cell-cycle dependent and mediated through phosphorylation (Lahav-Baratz et al., 1995; Visintin et al., 1997; Lukas et al., 1999; Kramer et al., 2000). CDH1 is inactivated through phosphorylation prior to metaphase onset, while Cyclin-dependent kinase (CDK) activity is high, and CDC20 is active during mitosis (Lukas et al., 1999; Kramer et al., 2000; Kernan et al., 2018). CDC20 recruitment to the APC/C is only allowed upon phosphorylation of APC1, which allows for APC3 to be phosphorylated and for the binding of the CDC20 IR tail (Kramer et al., 2000; Fujimitsu et al., 2016; Qiao et al., 2016; Zhang et al., 2016). CDH1, in turn, ensures CDC20 is inactivated in late mitosis-G1 through APC/C-dependent ubiquitination and through autoubiquitination (Visintin et al., 1997; Uzunova et al., 2012). In addition to the recognition, recruitment, and positioning of substrates near the active site, coactivator binding mobilizes the cullin-RING ligase (CRL) core for E2 recruitment and binding (Figures 2A,B; Yu et al., 1998; Tang et al., 2001; Chang et al., 2014, 2015; Li et al., 2016).

For substrate ubiquitination to occur, the APC/C utilizes a dual E2 mechanism where UBE2C initially primes the substrate with Ub and UBE2S extends K11-linked Ub chains (Sudakin et al., 1995; Aristarkhov et al., 1996; Yu et al., 1996; Garnett et al., 2009; Williamson et al., 2009; Wu et al., 2010; Wickliffe et al., 2011). In the complete absence of these well-established E2s, UBE2D/UBCH5 can also be used as the E2 (Wild et al., 2016). Detailed biochemical and structural studies have been performed to capture the multiple steps of ubiquitination by APC/C and its E2s. Upon CRL mobilization by the coactivators, UBE2C is grasped by the winged-helix B (WHB) and RING domains of APC2 and APC11, respectively, and positioned to transfer the Ub onto a substrate lysine (Figure 2C; Chang et al., 2014, 2015; Van Voorhis and Morgan, 2014; Brown et al., 2015; Li et al., 2016). In addition to substrate lysine modification, UBE2C can build short chains on substrate-linked Ub, catalyzing K11, K48, and K63 Ub chains (Figure 2D; Kirkpatrick et al., 2006; Dimova et al., 2012; Brown et al., 2015, 2016). Next, the Ub-conjugating domain (UBC) domain of UBE2S is activated for Ub transfer by a separate site on APC2, termed [UBE2S-interacting (Si) helices] (Brown et al., 2014, 2016; Kelly et al., 2014). Instead of binding and activating UBE2S, as in UBE2C, the APC11 RING domain accommodates the substrate-linked acceptor Ub to receive the Ub from UBE2S∼Ub (Figure 2E; Brown et al., 2014, 2016; Yamaguchi et al., 2016). To facilitate binding to the APC/C, UBE2S contains a C-terminal extension off its UBC that contains a positively charged peptide and binds in the groove formed between APC2 and APC4 (Williamson et al., 2009; Wickliffe et al., 2011; Chang et al., 2015; Brown et al., 2016; Yamaguchi et al., 2016).

Recent work has shown that the binding of the UBE2S C-terminal peptide (CTP) to the APC2/4 groove facilitates activation of the APC/C catalytic domain in a similar manner to coactivator binding to enhance the catalytic efficiency of UBE2C-dependent ubiquitination (Martinez-Chacin et al., 2020). Therefore, UBE2S facilitates a positive allosteric feedback mechanism to maximize substrate turnover by the APC/C. However, multiple questions remain about how the different APC/C subunits are repositioned for different modes of ubiquitination, how UBE2S is activated by the Si helices of APC2, and how branched Ub chains are formed.

While the substrate is still bound to the APC/C for Ub modification, the substrate-linked Ub has been shown to enhance processivity and substrate turnover rates (Lu et al., 2015; Brown et al., 2016). Ub binding to the APC11 RING domain, which positions the substrate-linked acceptor Ub for Ub-chain elongation by UBE2S, increases the binding affinity of the substrate and increases the processivity of the UBE2C-dependent reaction (Brown et al., 2016). A second, cryptic Ub-binding site was uncovered on the APC2 WHB using a tight-binding Ub variant (Figure 2F; Watson et al., 2019b). Interestingly, this binding site on the APC2 WHB is also utilized to position UBE2C and bind to a component of the inhibitory mitotic checkpoint complex (MCC). Therefore, this Ub-WHB interaction likely has multiple roles in regulating APC/C function during the cell cycle.

## The APC/C and Its Inhibitors

Canonical APC/C activity occurs in M and G1 phases, yet the APC/C is present throughout the cell cycle. The early mitotic inhibitor (EMI1) and the MCC both restrain APC/C function during the G1/S transition and the metaphase-anaphase transition, respectively (Hoyt et al., 1991; Li and Murray, 1991; Reimann et al., 2001b; Fang, 2002; Davenport et al., 2006; Miller et al., 2006; Burton and Solomon, 2007; Cappell et al., 2016). Both inhibitors attach to the APC/C at specific sites to selectively modulate substrate binding and Ub transfer by the E2s, and are regulated by ubiquitination-dependent degradation, releasing the APC/C from their inhibition.

### The Interphase APC/C Inhibitor EMI1

APC/C activity during interphase is regulated through several factors, including CDK-dependent phosphorylation, coactivator regulation, E2 degradation, and transcription of APC/C subunits (Kernan et al., 2018; Kataria and Yamano, 2019). One regulatory factor of note is EMI1, a protein that inhibits APC/C activity during G1/S phase transition to allow sufficient cyclin accumulation for mitotic entry (Reimann et al., 2001a,b). Since its discovery in *Xenopus* embryos, the significance of EMI1 has been elevated through numerous cell-cycle and cancer biology studies, including a live cell imaging study where EMI1 was identified as a key “point of no return” step for cell cycle reentry by inactivating the APC/C at the G1/S boundary (Di Fiore and Pines, 2008; Shimizu et al., 2013; Barr et al., 2016; Cappell et al., 2016; Guan et al., 2016; Vaidyanathan et al., 2016; Marzio et al., 2019; Moustafa et al., 2021). Therefore, several biochemical and structural studies have uncovered how EMI1 tightly binds and shuts down APC/C activity.

**FIGURE 3:**
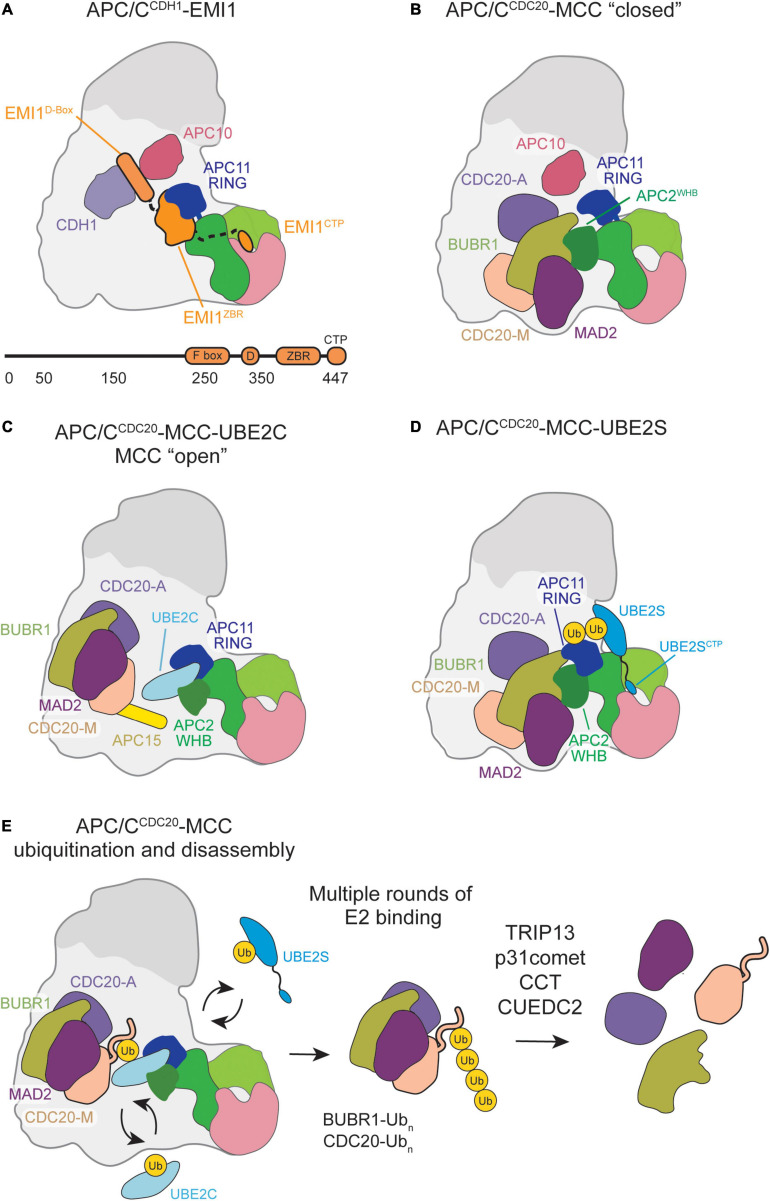
Schematic of APC/C inhibition by EMI1 or MCC binding. **(A)** EMI1 binds in a multimodal fashion, the D-box binds at the CDH1-APC10 D-box receptor site, binds and immobilizes the APC11 RING, and contains a CTP similar to the UBE2S CTP that binds in the APC2/4 groove. **(B)** MCC bound to the APC/C in a “closed” conformation engages the APC2 WHB and CDC20^A^. **(C)** MCC transitions to an “open” conformation, allowing UBE2C to bind, and some substrates, such as Cyclin-A and NEK2A, to bypass MCC inhibition and be ubiquitinated by the APC/C. **(D)** MCC in the “closed” conformation permits UBE2S to bind, allowing for Ub chain elongation to occur. **(E)** Multiple rounds of E2 binding result in the polyubiquitination of several MCC subunits. CCT/TRiC and TRIP13-p31^comet^ assist in the release CDC20 and MAD2, respectively, from MCC.

Multiple domains of EMI1 cooperate together to block multiple steps of the ubiquitination mechanisms (Figure 3A). Reimann et al. initially characterized the mode of inhibition by EMI1 by mapping its domains to their effects on APC/C activity (Reimann et al., 2001b). Although EMI1 is an F-box containing protein that is 50 kDa in size, only its C-terminus (16 kDa) contains the domains that are expected to function in APC/C inhibition (Reimann et al., 2001b; Frye et al., 2013; Wang and Kirschner, 2013). Within its C-terminus, EMI1 contains a D-box motif, linker, zinc binding region (ZBR), and a C-terminal peptide (Reimann et al., 2001b; Miller et al., 2006; Frye et al., 2013; Wang and Kirschner, 2013; Chang et al., 2015). The early studies of EMI1-mediated APC/C inhibition focused primarily on the D-box and ZBR (Reimann et al., 2001b; Miller et al., 2006). For example, EMI1 becomes a D-box-dependent APC/C^CDH1^ substrate upon ZBR inactivation, suggesting it functions as an APC/C^CDH1^ pseudo-substrate inhibitor (Miller et al., 2006). However, the intricate and specific mechanism was not fully appreciated yet, as UBE2S had not been identified as the chain-elongating E2.

Subsequent ubiquitination and structural studies expanded on this complex mechanism of E3 ligase inhibition by providing specific inhibitory roles for the individual domains. First, low-resolution EM structures demonstrated that the EMI1 D-box binds to CDH1 and APC10, while the ZBR and rest of the C-terminus was bound to the APC/C catalytic core and platform (Frye et al., 2013; Wang and Kirschner, 2013). Second, while the D-box of EMI1 does inhibit APC/C-dependent ubiquitination by itself, it is comparatively weak, suggesting that additional domains are needed to effectively inhibit the APC/C (Frye et al., 2013). Third, a linker between the D-box and ZBR was identified (Frye et al., 2013). Together with the ZBR, the linker contributes to EMI1-dependent inhibition of UBE2C-mediated monoubiquitination (Frye et al., 2013; Wang and Kirschner, 2013). Lastly, the EMI1 C-terminal tail was found to have a similar sequence to the UBE2S C-terminus and is sufficient to inhibit UBE2S-mediated Ub-chain elongation (Frye et al., 2013; Wang and Kirschner, 2013). Removal of this sequence significantly impairs the inhibition constant of EMI1. Taken together with additional NMR and other biophysical studies that suggest that the EMI1 C-terminal domain is largely disordered, except for the ZBR, these EMI1 domains synergize for strong APC/C^CDH1^ inhibition (Frye et al., 2013; Wang and Kirschner, 2013).

To fully understand the EMI1 inhibition mechanism, a high-resolution structure of the APC/C^CDH1^-EMI1 ternary complex was solved at 3.6 Å by cryo-EM (Chang et al., 2015). Through this structure, the multimodal APC/C^CDH1^-EMI1 interaction network was described in detail, and largely validated previous biochemical studies. As expected, the EMI1 D-box engages D-box receptors CDH1 and APC10 (Figure 3A). The EMI1 linker regions are packed against the ZBR β-sheet and bind the APC2-APC11 catalytic domain, effectively blocking UBE2C binding to APC11 and the RING domain. On the APC/C Platform, the EMI1 C-terminal peptide binds APC2 adjacent to the APC4 WD40 region, blocking UBE2S binding.

Despite this structural and mechanistic understanding, several biological implications have yet to be described. For example, EMI1 has been shown to be a substrate of SCF^βTRCP^ and the APC/C itself (Margottin-Goguet et al., 2003; Eldridge et al., 2006; Cappell et al., 2018). Further mechanistic studies are needed to describe how ubiquitination events occur and how these different domains are potentially regulated to selectively permit different APC/C ubiquitination mechanisms and/or strip this tight binding inhibitor off the APC/C.

### The Mitotic Checkpoint Complex

The spindle assembly checkpoint (SAC) prevents the cell from transitioning to anaphase prior to complete chromosome bi-orientation by regulating APC/C activity (Hoyt et al., 1991; Li and Murray, 1991; Fang, 2002; Davenport et al., 2006; Burton and Solomon, 2007). Chromosome kinetochores that remain unattached to the spindle apparatus signal the assembly of the MCC—a 250 kDa complex comprised of BUBR1, CDC20, BUB3, and MAD2 that binds and inhibits APC/C (APC/C^MCC^) (Hoyt et al., 1991; Li and Murray, 1991; Fang, 2002; Davenport et al., 2006; Burton and Solomon, 2007). The signaling networks and underlying mechanisms behind MCC assembly during SAC activation are reviewed in Musacchio (2015). Additionally, recent studies have uncovered novel, multiplex interactions that facilitate MAD2 binding to CDC20—the initial, rate-limiting step of MCC assembly —through *in vivo* and *in vitro* work (Lara-Gonzalez et al., 2021; Piano et al., 2021).

A number of pioneering structural studies worked to understand the basic structure of MCC and its inhibition of APC/C. In *Schizosaccharomyces pombe* MCC, MAD2, and MAD3 (BUBR1 in human MCC) were shown to cooperatively bind and inhibit CDC20 through multiplex interactions (Chao et al., 2012). CDC20 and MAD2 primarily interact through the MAD2 safety belt latching onto the CDC20 MAD2-interacting motif while MAD3 coordinates the overall structure of the complex (Fang et al., 1998; Luo et al., 2000, 2004; Sironi et al., 2002; Yu, 2006; Yang et al., 2007; Luo and Yu, 2008; Kim et al., 2014; Rosenberg and Corbett, 2015; Singh et al., 2017). Once assembled, MCC is capable of inhibiting APC/C activity through a “closed” conformation that was first observed through low-resolution single-particle EM of APC/C isolated from SAC-arrested cells (Herzog et al., 2009). In the “closed” conformation, MCC blocks the APC/C central cavity, preventing substrate and UBE2C recruitment. However, this structural model lacked the resolution necessary to map APC/C^CDC20^-MCC interaction networks. Questions remained concerning the mechanisms behind MCC-mediated APC/C inhibition and MCC departure from the APC/C during checkpoint silencing.

For years, it was unknown how MCC leaves in a manner that maintains APC/C-CDC20 association to allow rapid modulation of APC/C^CDC20^ activity in response to unattached kinetochores. Biological studies proposed that an MCC subcomplex comprised of BUBR1, BUB3, and CDC20 (BBC) was the main checkpoint effector. The BBC would negate the need to disrupt the complex CDC20-MAD2 interactions required for MAD2 departure and CDC20’s continued association to APC/C. In response, it was suggested that APC/C^MCC^ contains two CDC20 molecules, both bound by BUBR1 at either of its two KEN boxes (Primorac and Musacchio, 2013). Biochemical studies confirmed this hypothesis, which showed that recombinant MCC can bind a second CDC20 associated with the APC/C (Izawa and Pines, 2015). MCC contains a CDC20 molecule (CDC20^M^) that binds the BUBR1 KEN1-box. Through the BUBR1 KEN2-box, the MCC may associate with a CDC20 molecule bound to APC/C (CDC20^A^) as a coactivator. This APC/C^MCC^ binding configuration would be further confirmed and characterized by high-resolution structural studies mapping APC/C^MCC^ interaction and identifying novel conformational states that allow checkpoint silencing.

Through recombinant cryo-EM structures of APC/C^MCC^, MCC was shown to capture key interfaces and domains critical for APC/C activity in the “closed” conformation (Figure 3B; Alfieri et al., 2016; Yamaguchi et al., 2016). CDC20^M^ and CDC20^A^ interact through their WD40 domains. BUBR1 stabilizes this dual-CDC20 interaction by contacting both CDC20 subunits with its KEN1 box, KEN2 box, D-box, and tetratricopeptide repeat (TPR) domains. Such interactions disrupt the CDC20^A^ degron binding sites necessary for APC/C substrate recognition. In contrast, MAD2 solely interacts with MCC subunits BUBR1 and CDC20^M^, which potentially stabilizes their respective interactions with CDC20^A^. Additionally, MCC sterically blocks UBE2C binding within the APC/C central cavity predominantly through BUBR1, whose TPR domain directly interacts with the APC2^WHB^. Overall, key APC/C^MCC^ interactions in the “closed” conformation prevent substrate recognition and UBE2C ubiquitination activity to accomplish checkpoint-mediated anaphase inhibition.

Both high-resolution structural studies identified a previously undiscovered APC/C^MCC^ “open” conformation in which CDC20^A^ remains in contact with BUBR1 and CDC20^M^ to prevent substrate recognition (Figure 3C; Alfieri et al., 2016; Yamaguchi et al., 2016). However, the BUBR1^TPR^-APC2^WHB^ interface is disrupted and MCC is rotated away from the APC11 RING domain to allow UBE2C binding within the central cavity. During this conformational change, the APC15 N-terminal helix becomes ordered and makes key interactions with APC4 and APC5 to stabilize the “open” conformation (Uzunova et al., 2012; Alfieri et al., 2016; Yamaguchi et al., 2016). UBE2C binding to the “open” APC/C^MCC^ results in UBE2C active site positioning toward CDC20^M^, potentially facilitating ubiquitination necessary for MCC departure.

While UBE2C is impacted by the APC/C^MCC^ “closed”/“open” transition, UBE2S is not. Previous biochemical work showed that UBE2S escapes this SAC regulatory feature, which was hypothesized to be due to non-canonical UBE2S binding at an APC11 RING exosite (Brown et al., 2014, 2016; Kelly et al., 2014). APC/C^MCC^ structures crosslinked with a UBE2S-Ub variant (UBv) conjugate confirmed UBE2S placement adjacent to the APC2 and APC11 subunits away from the central cavity and MCC inhibition (Figure 3D; Yamaguchi et al., 2016).

Several proteins have been implicated in SAC silencing through facilitating MCC disassembly. The AAA-ATPase TRIP13, its binding partner p31^comet^, and the chaperonin CCT/TRIC facilitate disassembly of free MCC in a multistep process (Eytan et al., 2014; Wang et al., 2014; Miniowitz-Shemtov et al., 2015; Kaisari et al., 2017; Alfieri et al., 2018). P31^comet^ binds and recruits MCC to TRIP13 through the MAD2 subunit (Miniowitz-Shemtov et al., 2015; Alfieri et al., 2018). This recruitment allows TRIP13 to trigger a conformational change in MAD2, catalyzed through TRIP13 ATPase activity, and promote MAD2 dissociation from CDC20 (Miniowitz-Shemtov et al., 2015; Alfieri et al., 2018). CCT/TRIC works to further disassemble MCC subcomplexes lacking MAD2, thereby completing the disassembly pathways of free MCC (Kaisari et al., 2017). Whether these SAC silencing effectors also promote MCC departure from APC/C remains to be seen, though one silencing effector has been linked to APC/C-MCC disassembly. A biological study found that the CUE-domain protein CUEDC2 promotes the departure of MAD2 from APC/C^MCC^ through direct interactions with CDC20 (Gao et al., 2011). However, this functionality was discovered prior to our understanding that two CDC20 molecules exists in APC/C^MCC^, and there have yet to be biochemical or structural studies conducted to elucidate CUEDC2-mediated APC/C-MCC disassembly mechanisms. Therefore, much remains to be uncovered regarding the roles and mechanisms of SAC silencing effectors on APC/C^MCC^ regulation.

Though structurally resolved, the “closed” and “open” APC/C^MCC^ conformational dynamics during checkpoint silencing raise questions surrounding MCC departure. The “open” conformation is necessary for UBE2C-mediated ubiquitination to trigger MCC release and relieve APC/C inhibition, yet this conformation comprises only a small subset of the APC/C^MCC^ population in structural studies. Further work is required to determine the dynamics of this conformational change and how it may be influenced by effector proteins during checkpoint silencing to promote rapid APC/C activation. Additionally, the order in which MCC subunits dissociate from the APC/C and how various SAC effector proteins influence this process remains elusive (Figure 3E).

## Recent Studies Reveal How Certain Substrates Escape Mitotic Checkpoint Inhibition

While the SAC is capable of preventing most APC/C^CDC20^-mediated substrate degradation, there are APC/C substrates capable of bypassing this inhibitory mechanism. The privileged ubiquitination of Cyclin A and NEK2A during an active checkpoint presented a multitude of questions regarding what molecular and regulatory factors determine the timing of substrate ubiquitination (den Elzen and Pines, 2001; Hames et al., 2001; Hayes et al., 2006; Di Fiore and Pines, 2010; Wieser and Pines, 2015). Recent high-resolution structures of these two substrates bound to APC/C^MCC^ identified unique binding modes that proposed mechanisms by which Cyclin A and NEK2A are able to escape SAC regulation.

### Cyclin A

Cyclin A promotes microtubule detachment from kinetochores during prometaphase, allowing error correction during chromosome bi-orientation and faithful sister chromatid segregation (Kabeche and Compton, 2013). However, persistent cyclin A activity prevents complete bi-orientation, requiring cyclin A stability to be regulated for mitotic progression (den Elzen and Pines, 2001; Kabeche and Compton, 2013). Interestingly, Cyclin A degradation begins in prometaphase after Cyclin B-CDK2 activation in a proteasome- and APC/C-dependent manner, though the SAC is active (den Elzen and Pines, 2001).

Biochemical studies sought to understand how Cyclin A ubiquitination is allowed during an active checkpoint. Cyclin A binding to the APC/C was found to depend on several key interactions. First, Cyclin A associates with Cks, which binds phosphorylated sites on APC/C (Di Fiore and Pines, 2010). Once bound, the Cyclin A N-terminus binds the CDC20 WD40 domain regardless of MCC, and therefore, regardless of checkpoint activation (Di Fiore and Pines, 2010). However, the molecular mechanisms behind this competition and the role of Cks remained unknown in the absence of a structural view of APC/C^MCC^ bound to a CDK-Cyclin A-Cks complex.

**FIGURE 4:**
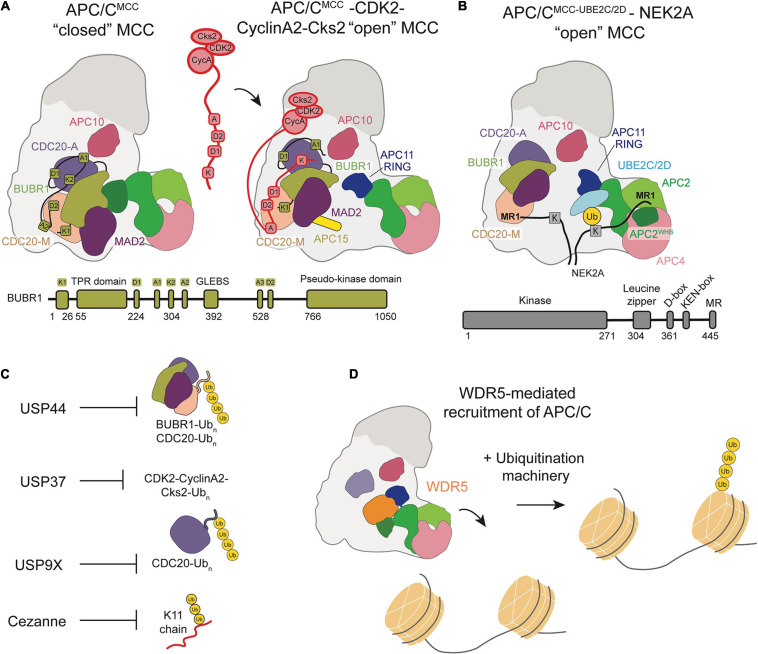
Schematic of APC/C regulation and chromatin regulation by the APC/C. **(A)** Model of the CDK2-Cyclin-A2-Cks2 complex bound to APC/C^MCC^ in the “open” conformation show how multiple Cyclin-A binding recognition motifs displace BubR1 binding motifs, allowing for Cyclin A to bypass MCC inhibition. **(B)** Depiction of NEK2A binding to APC/C^MCC^ “open” with NEK2A binding as a dimer and the MR1 motifs of each monomer binding either at APC8 or near the APC2/4 groove that binds the UBE2S^CTP^. **(C)** Summary of the DUBs that counteract APC/C ubiquitination throughout the cell cycle. **(D)** WDR5 recruits the APC/C to the nucleosome facilitating APC/C-mediated ubiquitination of histones.

Recently, a high-resolution structure of Cyclin A bound to APC/C^CDC20^, Cks, and CDK2 identified a non-canonical, highly conserved D-box (D2-box) on Cyclin A (Zhang et al., 2019). The canonical D1-box and non-canonical D2-box display differential binding with CDC20 and APC10, resulting in unique, cooperative interactions between the Cyclin A KEN box and ABBA motif and APC/C^CDC20^. Through its distinct binding mode, the Cyclin A D2-box directs more efficient Cyclin A ubiquitination than the canonical D1-box. A subsequent structure of APC/C^MCC^ bound to Cyclin A-CDK2-Cks2 proposed a mechanism by which Cyclin A circumvents MCC inhibition of the APC/C through multiple, disruptive interactions (Figure 4A). The Cyclin A D2-box and ABBA motif compete for CDC20^M^ binding with BUBR1^ABBA^. The Cyclin A KEN-box also competes with BUBR1^KEN^ for interactions with CDC20^A^, allowing Cyclin A to displace BUBR1 from APC/C^CDC20^. This cooperative interaction network is further stabilized by CDK and Cks, which bind both Cyclin A and phosphorylated APC/C sites.

Overall, the privileged ubiquitination of Cyclin A suggests the importance of substrate motifs and their avidity to the E3 ligase relative to regulatory factors in determining the timing and efficiency of substrate ubiquitination. Interestingly, Cyclin A was found to promote the APC/C^MCC^ “open” conformation, potentially to allow Cyclin A ubiquitination and degradation (Zhang et al., 2019).

### NEK2A

The kinetochore-associated NIMA-related kinase 2A (NEK2A) is able to evade APC/C inhibition by an active SAC to undergo ubiquitination and proteasomal degradation. Hames et al. first observed NEK2A degradation in early mitosis in an APC/C- and proteosome-dependent manner (Hames et al., 2001). Similar to canonical APC/C substrates, NEK2A contains a D-box and a KEN-box, as well as a suggested Cyclin A-like D-box motif at its extreme C-terminus. Domain-mapping biochemical studies proposed molecular mechanisms by which NEK2A ubiquitination and destruction may escape SAC-mediated inhibition of APC/C.

Though the KEN box and ABBA motif were found to contribute to NEK2A ubiquitination, two structural features proved to be essential for NEK2A interactions with APC/C. Biochemical studies largely focused on the NEK2A C-terminal motif, which contains a Met-Arg (MR) dipeptide that allows NEK2A to bind apo-APC/C, potentially through TPR domains on APC6 and APC8 (Hayes et al., 2006). This binding mode is in contrast to Cyclin A, which primarily interacts with CDC20. Additionally, NEK2A contains a leucine zipper region that allows dimerization and contributes to APC/C-recognition, though the mechanism of this contribution was unclear. Though NEK2A may bind apo-APC/C, its degradation is delayed until the arrival of CDC20 and is insensitive to the presence of MCC (Boekhout and Wolthuis, 2015). These observations lacked a structural view to propose a mechanism by which NEK2A is able to escape SAC regulation.

A recent study sought to determine how NEK2A binds APC/C^MCC^ for ubiquitination during an active checkpoint by generating a high-resolution structure of NEK2A-APC/C^MCC^ (Alfieri et al., 2020). Through refinement of previously determined 3D models, Alfieri et al. discovered that the CDC20^M^ IR tail dissociates from its APC8 binding site in the “open” APC/C^MCC^ conformation (Alfieri et al., 2016; Alfieri et al., 2020). This structural change would allow NEK2A to bind APC8 with its MR tail, in agreement with previous studies suggesting the importance of TPR-containing APC/C subunits (Figure 4B). As a dimer, NEK2A contains two MR tails. The binding site of the second MR tail was identified in a pocket formed by APC2^WHB^, APC2, and APC4^WD40^, potentially ordering the WHB domain to force an active APC/C configuration for NEK2A ubiquitination. Based on these observations, Alfieri et al. hypothesize that NEK2A promotes the “open” APC/C^MCC^ conformation by displacing the CDC20^M^ IR tail from APC8 and disrupting BUBR1-WHB binding. NEK2A is able to position the WHB domain 60 Å from its position in the “closed” conformation, prioritizing NEK2A ubiquitination rather than MCC-mediated inhibition.

Overall, current structural studies have uncovered the molecular mechanisms by which Cyclin A and NEK2A are able to evade MCC regulation and undergo APC/C-mediated ubiquitination. However, additional questions remain regarding interactions between these privileged substrates and E2 enzymes. It has been shown that NEK2A is more efficiently ubiquitinated by UBE2D than UBE2C, while the opposite is true for Cyclin A (Zhang et al., 2019; Alfieri et al., 2020). Further structural studies are needed to fully characterize substrate-E2 combinations. Together with current models of substrate-APC/C binding modes, such information will help uncover how the timing of substrate ubiquitination by the APC/C is controlled, with potential implications across E3 ligases.

## DUBS That Antagonize APC/C Function

The complexity of substrate ubiquitination and APC/C inhibition mechanisms are further compounded by the antagonism of deubiquitinase enzymes (DUBs). DUBs cleave Ub chains from substrates and can therefore prevent degradation. Four DUBs were identified to specifically antagonize APC/C-mediated ubiquitination and degradation of substrates: USP44, USP37, USP9X, Cezanne/OTUD7B (Figure 4C). These DUBs work in opposition to the APC/C to regulate cell cycle progression.

Drugs that disrupt the mitotic spindle, including taxol, nocodazole and vincristine, disrupt kinetochore-microtubule attachments and maintain active spindle checkpoint signaling, thus restraining APC/C activation. In a search for DUBs that might regulate cell division, Stegmeier et al. (2007) used RNAi to screen for genes whose loss caused a bypass of mitotic arrest in the presence of taxol. This analysis identified the ubiquitin specific protease, USP44. They showed that USP44 antagonizes the ubiquitination of the MCC (Figure 4C). Importantly, MCC ubiquitination leads to spindle checkpoint silencing. Thus, in the absence of USP44, MCC ubiquitination is increased and the ability of the complex to restrain cell division is lost. This study represented the first identification of a DUB linked to APC/C function.

Despite the ability of EMI1 to potently inhibit interphase APC/C, it was later noted that not all APC/C^CDH1^ is associated with EMI1 during G2 phase. This raised the question as to how APC/C substrates remain stable prior to mitosis. Huang et al. hypothesized that an additional mechanism of APC/C^CDH1^ inactivation functions to prevent degradation of APC/C^CDH1^ substrates after G1 phase (Miller et al., 2006; Bassermann et al., 2008; Huang et al., 2011). Specifically, the presence of a DUB could prevent degradation of APC/C substrates while maintaining a pool of APC/C available to be activated. They identified interactions between USP37 and CDH1, as well as APC/C subunits, implicating USP37 in the regulation of the G1/S transition and characterized the cell cycle regulation of USP37 (Figure 4C). Based on this study, Huang et al. describe a model where USP37 is transcribed in late G1 and the resulting protein antagonizes APC/C-mediated ubiquitination of Cyclin A, resulting in accumulation of Cyclin A which then promotes progression to S phase (Huang et al., 2011). Cyclin A accumulation activates CDK2, which also phosphorylates USP37 to positively reinforce its catalytic activity. As cells progress to mitosis, Cyclin A is inactivated and APC/C is activated, at which point USP37 switches from an antagonist of APC/C ubiquitination and is itself ubiquitinated by the APC/C and degraded. This prevents USP37 from antagonizing APC/C substrate degradation. This study demonstrates the role of a DUB in regulating the G1/S transition by opposing APC/C activity through its interaction with Cyclin A. USP37 has also been linked to the regulation of other cell cycle proteins, including Cdt1 and WAPL (Yeh et al., 2015; Hernandez-Perez et al., 2016). Interestingly, USP37 is also a substrate of SCF-type ubiquitin ligases, and this too is cell cycle regulated (Burrows et al., 2012).

The role of the APC/C in promoting progression from metaphase to anaphase during mitosis was also found to be antagonized by the DUB USP9X. Skowyra et al. (2018) showed that USP9X strengthens the SAC by antagonizing APC/C ubiquitination of CDC20, which represents a critical point of regulation to prevent chromosomal instability. During mitosis, the SAC prevents progression to anaphase until all chromosomes are properly attached to microtubules. Until this occurs, the MCC is continuously assembled, of which CDC20 is a component (Lischetti and Nilsson, 2015; Musacchio, 2015). APC/C-mediated CDC20 autoubiquitination results in MCC turnover (Uzunova et al., 2012; Izawa and Pines, 2015; Alfieri et al., 2016; Yamaguchi et al., 2016). The synthesis of new MCC compensates for MCC turnover until all chromosomes are prepared for anaphase. This regulation is very sensitive, although the molecular mechanism for the sensitivity remained unknown until Skowyra et al. investigated the possibility that a DUB opposing APC/C activity could contribute to this phenomenon (Skowyra et al., 2018). They demonstrated that USP9X depletion causes premature mitotic progression in the presence of the microtubule poison nocodazole and that it antagonizes APC/C-mediated ubiquitination of CDC20 (Figure 4C). Additionally, they showed that USP9X depletion increases the degradation of CDC20 and leads to bypass of SAC arrest, resulting in chromosomal instability. Thus, USP9X plays an important role in regulating appropriate chromosome segregation and genome stability during mitosis by antagonizing ubiquitination by the APC/C.

An additional DUB opposing ubiquitination by the APC/C was identified by our labs (Bonacci et al., 2018). In a search for K11 linkage specific DUBs, it was confirmed that Cezanne/OTUD7B specifically cleaves K11-linked ubiquitin chains. Notably, Cezanne is itself a strongly cell cycle regulated (Figure 4C; Bremm et al., 2010; Mevissen et al., 2016). Our work demonstrated that Cezanne levels peak during mitosis, in concert with APC/C activity, and that Cezanne specifically interacts with, deubiquitinates, and opposes the degradation of APC/C substrates, including Aurora A and Cyclin B (Bonacci et al., 2018). The functional consequence of Cezanne activity was shown by experiments in which Cezanne depletion resulted in mitotic progression errors and micronuclei formation. This study, along with those previously discussed, indicate key roles for DUBs that specifically oppose APC/C-mediated ubiquitination in regulating proper cell cycle progression. Future studies may identify additional roles and substrates of cell cycle-regulated DUBs. Determining how these DUBs, and perhaps others, coordinate with each other to regulate the kinetics of substrate degradation represents an important area of future investigation.

## APC/C Regulates Chromatin

Chromatin experiences many dynamic changes during the cell cycle, most notably genome replication during S phase and chromosome condensation and segregation during mitosis. Additionally, subsets of genes are transcribed in a cell cycle-dependent manner (Whitfield et al., 2002; Bar-Joseph et al., 2008; Grant et al., 2013; Pena-Diaz et al., 2013). The promoters and enhancers of these genes must be available for binding by transcriptional machinery which requires changes in chromatin structure at these loci during the cell cycle. While aspects of chromatin organization are coordinated with cell cycle progression, the APC/C was only recently identified to play a role in this regulation. The following studies demonstrated direct interactions between the APC/C and nucleosomes, as well as with DNA itself. These observations show new biological functions of APC/C and raise additional questions about the interactions between chromatin and the APC/C.

The fundamental unit of chromatin structure is the nucleosome, consisting of a core octameric subunit of histone proteins around which the DNA double-helix is wound (Luger et al., 1997). Nucleosomes play a role in compacting DNA, but also serve as a key point of signal integration, as many proteins interact with various components of the nucleosome structure to affect local genome accessibility, among other functions (Kouzarides, 2007; Struhl and Segal, 2013). As part of a study to identify the nucleosome interaction network and establish principles for nucleosome-binding proteins, Skrajna et al. (2020) observed multiple protein components of the APC/C bound to the nucleosome by mass spectrometry. Direct binding of the APC/C to the nucleosome was also established, suggesting the possibility that the APC/C may play a fundamental role in nucleosome ubiquitination.

Oh et al. (2020) published a study at a similar time demonstrating a functional role for the APC/C at nucleosomes by showing the ubiquitination of histones by the APC/C in human embryonic stem cells. They identified the APC/C as a potential integrator of cell division and the pluripotency transcriptional program. This transcriptional program is essential to maintaining stem cell identity, but transcription is generally downregulated during mitosis, bringing into question the mechanism by which cells are able to transcribe pluripotency factors immediately following cell division to maintain stem cell identity (Prescott and Bender, 1962; Young, 2011). Oh et al. (2020) demonstrated that the chromatin-associated factor WDR5 recruits the APC/C to the promoters of pluripotency genes marked by stem cell specific transcription factors during mitosis. K11/K48 branched ubiquitin chains, a hallmark of APC/C function, on histones were identified at these promoter regions and shown to be targeted for degradation by the proteasome. Based on this study, they proposed a mechanism in which WDR5 binds promoters of pluripotency genes during interphase and remains bound as cells enter mitosis, at which point the APC/C is recruited to transcription start sites and ubiquitinates histones (Figure 4D). An EM structure of WDR5 bound to the APC/C revealed that WDR5 is bound to the catalytic core APC2-APC11. This structure suggests that WDR5 would have to leave the APC/C for APC/C and its E2s to ubiquitinate the nucleosome, or another unexpected catalytic architecture must be formed. After ubiquitination, the histones are degraded by the proteasome to maintain an open chromatin structure at gene promoters, allowing the transcription of pluripotency genes immediately after the completion of mitosis. This study characterizes a functional role of APC/C interaction with nucleosomes, implicating the APC/C as a regulator of chromatin organization and pluripotency.

An additional study by Mizrak and Morgan (2019) implicated binding of the APC/C to polyanions, including nucleic acid polymers which are components of nucleosomes, as a mechanism to regulate the dissociation of CDC20 and CDH1 from the APC/C. Using lysates from *Saccharomyces cerevisiae* to perform *in vitro* experiments, they show that single-stranded DNA and RNA of about 75 base pairs, as well as polyphosphate species, promote the dissociation of CDC20 and CDH1 from the APC/C. However, the polyanions lose their ability to promote coactivator dissociation when the APC/C is bound to a substrate with high affinity for the complex. Their proposed mechanism described the interaction between the APC/C and polyanions as a way to promote ubiquitination of high-affinity substrates, while reducing the ubiquitination of low-affinity substrates by causing the dissociation of the coactivator from the APC/C. From this conclusion, they hypothesized that polyanion binding could interact with the APC/C at sites adjacent to the coactivators. However, additional studies are required to validate this hypothesis. Confirmation of this model would indicate a new functional interaction between the APC/C and nucleic acids, suggesting that coactivator dissociation could be another point of regulation of APC/C activity and affect how the DNA-wrapped nucleosome is ubiquitinated.

The APC/C was further implicated in the regulation of chromatin biology with the identification of novel APC/C substrates (Franks et al., 2020). Franks et al. (2020) used an *in silico* approach to identify novel APC/C^CDH1^ substrates based on two criteria shared by many known APC/C^CDH1^ substrates. The first was the presence of a KEN-box degron. The second criterion was that the gene encoding for the protein is transcribed in a cell cycle-dependent manner, as identified in cell cycle transcriptome studies, based on RNA-sequencing and microarrays. These criteria identified 145 proteins, including many previously identified APC/C^CDH1^ substrates. The resulting candidate substrates were enriched for GO processes related to chromatin biology. Several candidates were shown to oscillate during the cell cycle, to be degraded when the APC/C is activated, and to co-immunoprecipitate with CDH1, including histone modification writers UHRF1 and TTF2, and the chromatin assembly factor CHAF1B. The ubiquitination of these proteins was previously reported in independent studies (Kim et al., 2011; Elia et al., 2015). We further characterized the mechanism and functional consequence of degradation of UHRF1, a chromatin regulator involved in histone ubiquitylation and maintenance of DNA methylation (Bostick et al., 2007). We demonstrated that disruption of UHRF1 degradation by the APC/C at mitotic exit results in altered DNA methylation patterns across the genome and accelerated progression through G1 phase. This study describes several novel APC/C substrates involved in the regulation of chromatin biology and shows that proper degradation of UHRF1 is important for chromatin biology and for cell cycle progression.

These recent studies indicate that the APC/C regulates aspects of chromatin biology in addition to its role in promoting mitotic progression. Cyclin-dependent kinases (CDKs) were also recently implicated in regulation of chromatin biology with the identification of novel CDK substrates responsible for regulating the epigenetic landscape (Chi et al., 2020; Michowski et al., 2020). Thus, it is likely that chromatin biology is broadly regulated by multiple components of the cell cycle machinery. Future work may identify additional enzyme-substrate relationships connecting these two areas of biology and may elucidate the functional consequences of these interactions.

## Discussion

Understanding how cells orchestrate a delicate balance between protein accumulation and degradation remains a significant challenge. Various rules have been suggested previously. For example, processive substrates, substrates that are highly ubiquitinated in a single binding event, are degraded faster when compared to distributive substrates, i.e., substrates that require multiple binding events to build a proteasome degradation signal (Rape et al., 2006; Buschhorn et al., 2011; Meyer and Rape, 2011; Williamson et al., 2011; Lu et al., 2015). However, the molecular description of this rule and others remains largely uncharacterized. Key questions remain: What makes an APC/C substrate processive—cooperativity between the degrons, lysine accessibility, catalytic rate of ubiquitination, or a combination? How do multiple ubiquitination mechanisms synergize for accurate cell cycle timing? Given the diversity of APC/C substrates, additional mechanisms are likely to be uncovered. For example, we likely do not know if a certain set of substrates is critically dependent on UBE2S or how the ubiquitination mechanism is altered for the autoubiquitination of CDH1 and its E2s. In addition to post-translational modifications, e.g., phosphorylation and sumoylation, on the APC/C, substrates can also be phosphorylated to alter their degradation rate, but the mechanistic basis for this regulation is unknown (Min et al., 2013; Lu et al., 2014; Davey and Morgan, 2016; Eifler et al., 2018; Lee et al., 2018). To further complicate this process, DUBs fine tune the ubiquitin code by editing or completely removing Ub chains, extending the lifetime of a protein. The ∼100 DUBs can vary dramatically in their mechanism, Ub linkage specificities, cellular localization, post-translational modifications, and regulation (Mevissen and Komander, 2017). However, how specific DUBs are cell-cycle regulated or specifically antagonize APC/C function remains unclear.

The timing of cell cycle events is directly coupled to changes in the transcription of several hundred genes by chromatin regulation (Bannister and Kouzarides, 2011; Struhl and Segal, 2013; Ma et al., 2015). Moreover, the chromatin landscape is broadly altered during cellular quiescence, a reversible state of growth arrest and terminal differentiation (Buttitta et al., 2010; Evertts et al., 2013; Ma et al., 2015, 2019). However, the mechanisms underlying these dynamics are largely unknown. The recent data discussed above, and other previous studies, suggest that the APC/C is a significant regulator of cell cycle transcription and chromatin changes. First, the APC/C regulates cell cycle transcription factors, namely FOXM1 and E2F1 (Laoukili et al., 2008; Park et al., 2008; Peart et al., 2010; Ping et al., 2012). Second, the APC/C controls the levels of cell cycle transcriptional repressors, most notably, the atypical repressors E2F7 and E2F8 (Cohen et al., 2013; Boekhout et al., 2016). Third, the APC/C ubiquitinates chromatin modifying enzymes, including UHRF1 (Franks et al., 2020). Lastly, the APC/C directly ubiquitinates histones (Oh et al., 2020). Together, this places the APC/C at the center of proliferative control via the coordination of chromatin dynamics and gene expression.

These observations support the notion that activation of the APC/C acts as a molecular reset switch for proliferative transcriptional programs. APC/C activation can be thought of as the final weight, that when added to a scale, brings the cell back to a point where several proliferative signals are near zero. This reset happens through the inactivation of mitotic CDKs, as well as inactivation of transcriptional and chromatin programs (Guardavaccaro and Pagano, 2006; Skaar and Pagano, 2008; Buttitta et al., 2010; Ma et al., 2015, 2019). Interestingly, we normally consider these changes as being governed by the retinoblastoma (RB) family of transcriptional repressors (Guardavaccaro and Pagano, 2006). However, expression of the APC/C substrate UHRF1 in G1 phase, using a degradation-resistant allele, increases the expression of E2F targets, including cyclin E and E2F1 (Franks et al., 2020). These observations suggest that the regulation of chromatin proteins by APC/C-mediated destruction is indirectly linked to the expression of cell cycle transcriptional programs. Furthermore, previous studies showed that a non-degradable FOXM1 protein is sufficient to promote S-phase entry (Laoukili et al., 2008; Park et al., 2008). Together, these observations suggest that the destruction of many substrates by the APC/C is necessary to restrain the cell cycle. These findings are consistent with the significantly shortened G1-phase observed in CDH1-KO cells and the ability of CDH1 to suppress tumorigenesis in mice (Garcia-Higuera et al., 2008; Sigl et al., 2009).

There is significant cross talk between RB and APC/C pathways in restraining G1/S (Guardavaccaro and Pagano, 2006; Kernan et al., 2018; Emanuele et al., 2020). This relationship is particularly evident in the regulation of SKP2, which is both an E2F target gene and an APC/C substrate (Carrano et al., 1999; Tsvetkov et al., 1999; Bornstein et al., 2003; Bashir et al., 2004; Assoian and Yung, 2008; Yuan et al., 2014), In this example, the accumulation of SKP2 leads to the destruction of cyclin-dependent kinase inhibitors (CKIs), resulting in the activation of cyclin E/CDK2 complexes that drive S-phase entry through the phosphorylation of RB. The additional findings discussed above suggest that there are many more ways that the APC/C is regulating the transcriptional dynamics of the cell cycle, and we speculate that these pathways are deeply intertwined. Furthermore, the regulatory systems surrounding the APC/C, which involve kinases and DUBs, are likely to tune substrate ubiquitination, thereby shaping the chromatin environment and transcriptional programs. These relationships are likely to be highly relevant to cell cycle progression, and to quiescent or differentiated cells where the APC/C is also active.

The newfound relationship between the APC/C and chromatin may also play a significant role in tissue specific functions as genetic disorders are beginning to be found from the disruption of the APC/C function (Eguren et al., 2011; Huang and Bonni, 2016). For example, a mutation was found in CDH1 that causes neurological defects, e.g., microencephaly and epilepsy (Rodriguez et al., 2019). In another study, decreased *ANAPC1* transcript and corresponding APC1 protein levels results in Rothmund-Thomson syndrome that effects multiple organ systems (Ajeawung et al., 2019). Deeper genomic studies will likely demonstrate other functions and diseases genetically linked to the APC/C and its role in regulating chromatin. Mechanistically, it remains unclear why the APC/C specifically acts on chromatin regulators in G1, how the APC/C ubiquitinates the nucleosome, or how APC/C activity is regulated by negatively charged polyanions, such as nucleic acids. Additional studies will hopefully shed light on this relatively new and exciting era of APC/C-dependent biology and mechanisms.

## Author Contributions

TB, KW, MH, ME, and NB wrote and edited the manuscript. All authors contributed to the article and approved the submitted version.

## Conflict of Interest

The authors declare that the research was conducted in the absence of any commercial or financial relationships that could be construed as a potential conflict of interest. The reviewer MS declared a past collaboration with one of the authors ME to the handling editor.
